# Modification Approaches to Enhance Dehydration Properties of Sodium Alginate-Based Pervaporation Membranes

**DOI:** 10.3390/membranes11040255

**Published:** 2021-04-01

**Authors:** Mariia Dmitrenko, Vladislav Liamin, Anna Kuzminova, Erkki Lahderanta, Nikolay Solovyev, Anastasia Penkova

**Affiliations:** 1 St. Petersburg State University, 7/9 Universitetskaya nab., St. Petersburg 199034, Russia; lyamin.vlad.322@gmail.com (V.L.); ai.kuzminova@mail.ru (A.K.); a.penkova@spbu.ru (A.P.); 2 Laboratory of Physics, Lappeenranta University of Technology, Box 20, 53851 Lappeenranta, Finland; erkki.lahderanta@lut.fi; 3 Institute of Technology Sligo, Ash Lane, F91 YW50 Sligo, Ireland; solovyev.nikolay@itsligo.ie

**Keywords:** sodium alginate, substrates, fullerene derivatives, layer-by-layer assembly, polyelectrolytes, pervaporation dehydration

## Abstract

Transport characteristics of sodium alginate (SA) membranes cross-linked with CaCl_2_ and modified with fullerenol and fullerene derivative with L-arginine for pervaporation dehydration were improved applying various approaches, including the selection of a porous substrate for the creation of a thin selective SA-based layer, and the deposition of nano-sized polyelectrolyte (PEL) layers through the use of a layer-by-layer (Lbl) method. The impacts of commercial porous substrates made of polyacrylonitrile (PAN), regenerated cellulose, and aromatic polysulfone amide were investigated by scanning electron microscopy (SEM), atomic force microscopy (AFM), standard porosimetry method, and water filtration. The effects of PEL combinations (such as poly(sodium 4-styrene sulfonate) (PSS)/SA, PSS/chitosan, PSS/polyacrylic acid, PSS/poly(allylamine hydrochloride)) and the number of PEL bilayers deposited with the Lbl technique on the properties of the SA and SA/fullerene derivative membranes were studied by SEM, AFM, and contact angle measurements. The best characteristics were exhibited by a cross-linked PAN-supported SA/fullerenol (5%) membrane with five PSS/SA bilayers: permeation flux of 0.68–1.38 kg/(m^2^h), 0.18–1.55 kg/(m^2^h), and 0.50–1.15 kg/(m^2^h), and over 99.7, 99.0, and 89.0 wt.% water in the permeate for the pervaporation dehydration of isopropanol (12–70 wt.% water), ethanol (4–70 wt.% water), and tetrahydrofuran (5.7–70 wt.% water), respectively. It was demonstrated that the mutual application of bulk and surface modifications essentially improved the membrane’s characteristics in pervaporation dehydration.

## 1. Introduction

Surface processing for the acquisition of required properties of materials is in high demand in all fields of science and industry. In engineering, coatings are used to enhance corrosion resistance properties [1,2,3,4,5,6], to ensure thermally modified wood stability [7], and to improve the performance and lifespan of machining tribological tools [8]. Other applications may include the removal of dyes from wastewater [9], as well as vast usage in the biomedical industry [10] including corrosion protection for stainless steel substrates [11], and for the production of orthopedic and craniofacial implant devices [12], etc. Membrane technologies may be attributed to sustainable processes due to their valuable characteristics, including their environmental friendliness, low energy consumption, reagent-free composition, compactness in terms of equipment, and ease of automation. These advantages have ensured the decisive role of these technologies in the separation processes of various industrial fields (petrochemical, medical, biochemical, etc.) [13,14]. Surface coatings may be used in membrane technologies to improve pore clogging and antibacterial properties of membranes, to overcome fouling or improving solvents resistance, and to enhance membrane efficiency [15,16,17,18,19], while synthesizing brand new materials takes far more time, risk, and cost [20]. In particular, this direction of surface modification (coating) is actively developed and applied for the membranes used in pervaporation [20,21,22,23].

Pervaporation is one of the advanced membrane methods applied for the separation of low molecular weight component liquid mixtures. Compared to traditional separation methods (for example, distillation), it has significant advantages in the separation of azeotropic and isomer mixtures, and close-boiling and thermally unstable substances. Pervaporation offers high separation selectivity, without the use of an additional separating agent, with low energy consumption. The primary reason for this is that pervaporation separation is mainly based on the “solubility-diffusion” mechanism and is not limited by the vapor-liquid equilibrium of the system [24]. However, it is more cost-effective and more beneficial to use pervaporation in a combined (hybrid) process with conventional methods rather than an independent process. Such an approach increases productivity by removing the use of intermediate reagents, which require an additional purification stage [25]. In particular, pervaporation is widely used for alcohol and solvent dehydration [25,26]. However, significant insights are still required to improve the performance of pervaporation membranes, to reduce their cost, and to avoid defects in full-scale production. The most perspective and simple methods of surface modification of developed membranes are the deposition of polyelectrolytes (PEL) and thin selective layers (development of supported membranes), which significantly mediate the transport characteristics of the membranes [27,28,29,30,31,32].

One of the methods used to increase membrane efficiency for industrial applications is the development of a supported membrane, i.e., the coating of a porous substrate with a selective thin polymer layer. The structure and properties of the porous substrate largely determine the transport characteristics of the thin selective polymer layer [27,28]. The selection of a relevant porous support is necessary to form the thinnest possible selective layer of a supported membrane with good adhesion, enhancing the performance and providing mechanical strength for the thin layer.

To further improve the supported membrane performance, one of the most promising methods of surface modification is the deposition of nano-sized layers on the polymer layer surface [33,34]. This may be easily achieved by a simple, modern, and cost-effective layer-by-layer (Lbl) technique [35,36,37,38]. Rapid developments of the Lbl method have included the deposition of PEL on a film, providing tailored characteristics. The surface charge of PEL layers makes them attractive for the functionalization and coating of pervaporation membranes [39]. The deposition of PEL layers provides unique properties to the membrane surface. The formation of a charged film surface with high hydrophilicity results in a strong affinity for water, which directly affects the dehydration process. It is also worth noting that it is possible to achieve the desired transport characteristics of pervaporation membranes and to increase their efficiency and productivity for the dehydration process using this method [27,40,41]. The means to do so include the variation of types of PEL pairs, the number of deposited PEL nanolayers, ionic strength, pH, etc. [42,43,44].

The majority of previously published studies are devoted to the deposition of PELs on porous membranes (substrates) to create pervaporation multilayer membranes with ultra-thin selective layers. A single bilayer of branched polyethyleneimine (BPEI)/polyacrylic acid (PAA) was deposited on a hydrolyzed polyacrylonitrile (HPAN) hollow fiber membrane to prepare a composite hollow fiber membrane for the pervaporation separation of an ethanol/water mixture [29]. A PAN hollow fiber membrane (porous substrate) was hydrolyzed through immersion in NaOH aqueous solution at 50 °C before Lbl deposition. The NaOH treatment was carried out to induce the negative charge and increase the surface hydrophilicity, which was important for the subsequent Lbl coating of PEL and significantly influenced the membrane performance. The best transport properties (99 wt.% water content in the permeate and 0.233 kg/(m^2^ h) permeation flux) in the pervaporation dehydration of ethanol (10 wt.% water) at 25 °C were obtained for a PAA/BPEI/HPAN composite hollow fiber membrane with deposited 0.25 wt.% PAA solution and 0.1 wt.% BPEI solution on the surface of an HPAN substrate hydrolyzed by 2 mol/L NaOH at 50 °C. Multilayer PEL membranes were prepared using the Lbl method with the deposition of polyethylenimine (PEI)/polyacrylic acid (PAA) on an interfacially polymerized polyamide (PA) substrate for the pervaporation dehydration of ethylene glycol, ethanol, and isopropanol [30]. To stabilize the PEI/PAA membrane in the solvents (ethanol and isopropanol) and to improve the membrane selectivity, PEI was substituted with partially protonated chitosan (CS) in the last few PEL bilayers during the preparation of the membrane. It was demonstrated that using a porous polyamide (PA) membrane as a substrate made the production of pervaporation membranes from less than eight PEL bilayers possible, used for the dehydration of alcohols and a diol. Tannic acid (TA) and PEI were alternatively deposited on an hydrolyzed polyacrylonitrile (HPAN) porous membrane (substrate) via the Lbl method to obtain membranes with an ultrathin active layer for the pervaporation dehydration of ethanol [31]. The effects of pH, operation temperature, bilayer number, and water content in the feed on the membrane performance were investigated. In this study, a PAN membrane (substrate) was also hydrolyzed in NaOH solution (1.5 mol/L) at 55 °C for 1 h to achieve increased surface hydrophilicity and to improve the adhesion of PEL. A membrane with 5.5 PEI/TA bilayers prepared at pH 8 exhibited the optimal transport characteristics in the pervaporation dehydration of ethanol (10 wt.% water) at 76 °C: 1.343 kg/(m^2^ h) and separation factor of 1012, due to the increased membrane surface hydrophilicity and the favorable free volume of thin PEL layers. In the work [45], composite PEL pervaporation membranes were prepared through the Lbl deposition of PEI/poly(4-styrene sulfonic acid-co-maleic acid) (PSSMA) on a porous asymmetric modified PAN (mPAN) substrate. The PAN substrate was also subjected to preliminary hydrolysis (through immersion in 2 mol/L NaOH aqueous solution at 50 °C for 10 min) so that the surface nitriles were converted to carboxyl groups. The membrane prepared from 1 bilayer of 0.9 wt.% aqueous PEI solution and 0.1 wt.% aqueous PSSMA solution on the mPAN substrate (contacting with PEL for 15 min) demonstrated the best transport properties in the pervaporation of 90 wt.% alcohol/water solutions at 25 °C. The results were as follows: 44.7, 89.6, and 90.8 wt.% water content in the permeate and 695, 645, and 348 g/(m^2^ h) permeation flux for methanol, ethanol, and isopropanol dehydration, respectively. These studies demonstrate a promising application of PEL bilayers for the preparation of pervaporation membranes with improved performance. Additionally, the studies indicate that the selection of a porous substrate and its properties largely affected the characteristics of the thin layer deposited on it.

Despite the promising use of the Lbl method for the deposition of PEL demonstrated for porous substrates, there is only a limited number of works in which PEL is applied for coating and modifying pervaporation membranes (with a top non-porous layer) [27,32,40,41]. In the work [32], the transport properties of a hybrid polyvinyl alcohol (PVA)-tetraethyl orthosilicate (TEOS) membrane for pervaporation dehydration were improved by applying the layer-by-layer deposition of polyvinyl amine (PVAm)/silicotungstic acid (STA) polyelectrolytes. It was demonstrated that the coating of five PVAm/STA bilayers on a PVA/TEOS membrane was optimal for surface modification, increasing the permeation flux from 0.14 to 0.22 kg/(m^2^ h) and decreasing the separation factor from 2099 to 1100 in the pervaporation dehydration of an epichlorohydrin/isopropanol/water (50/30/20 wt.%) mixture compared to the unmodified PVA/TEOS membrane. The increased permeation flux was attributed to the formation of small hydrophilic mashes induced by the high charge density of PEL, a layer favoring water penetration. The increased hydrophilicity of the membrane surface caused the enhanced swelling of the membrane surface promoting mutual epichlorohydrin/isopropanol absorption with water resulting in a decreased separation factor.

Our previous works [27,40,41] explored the development of supported membranes and PEL deposition by the Lbl technique. We demonstrated that the surface modification of polyvinyl alcohol (PVA)-based membranes led to enhanced pervaporation dehydration. In the work [27], the impact of porous PAN and aromatic polysulfone amide (UPM) substrates was investigated in the supported PVA membranes. It was demonstrated that the deposition of a thin PVA-based layer on the PAN substrate increased the selective properties and decreased the permeation flux (~4 times) in the pervaporation dehydration of isopropanol (20 wt.% water), compared to the use of the UPM substrate. It is also worth noting that surface modification, namely the deposition of ten bilayers of poly(allylamine hydrochloride)/poly(sodium 4-styrene sulfonate) (PAH/PSS) by Lbl assembly, of supported PVA-based membranes causes them to act completely differently depending on the substrate used. The PVA/PAH (4.7%) membrane on the PAN substrate modified with ten PSS/PAH bilayers possessed the optimal transport characteristics in the pervaporation dehydration of isopropanol (20 wt.% water): 0.061 kg/(m^2^ h) permeation flux and 99.9 wt.% water in the permeate.

In our earlier work [41], simultaneous bulk (the introduction of fullerenol and PAH) and surface (Lbl assembly for PEL deposition) modifications of PVA membranes were applied to create mixed matrix membranes with improved transport characteristics for pervaporation dehydration. The Lbl deposition of ten bilayers of PAH/PSS on the surface of PVA-supported membranes on a UPM substrate led to an increase of permeation flux. However, it also led to a significant decrease in selectivity, while the combination of bulk and surface modification significantly increased the permeation flux, maintaining a high water content in the permeate (over 98%). The supported membrane with a thin selective layer based on a composite PVA/fullerenol (5%)/PAH (4.7%)/maleic acid (35%) deposited on a UPM substrate with surface modification with a ten PSS/PAH bilayer coating had the best transport properties in the pervaporation dehydration of isopropanol (20 wt.% water): 98.4 wt.% water in the permeate and 0.286 kg/(m^2^ h) permeation flux, which was 8.5 times higher compared to the commercial analog membrane, PERVAP^TM^ 1201 (Sulzer Chemtec Co., Allschwil, Switzerland).

In both studies [27,41], polyelectrolyte PAH was introduced into the PVA matrix to improve the dispersion of fullerenol in the PVA matrix, improving the membrane’s selectivity. The effect of PAH is related to its high affinity for water, as well as its capability to facilitate the adhesion of PEL layers during surface modification. A novel supported PVA membrane with improved transport properties was developed through the bulk (blending with CS and the introduction of fullerenol) and surface (the development of a supported membrane on a UPM substrate and Lbl coating of nano-sized PEL layers) modification in the study [40]. It was demonstrated that a suitable PEL pair, the order of PEL deposition, and the number of PEL bilayers, as well as bulk modifiers (CS and/or fullerenol), had a significant effect on the membrane performance. The best transport properties for the isopropanol dehydration (20 wt.% water) were exhibited by a membrane based on a PVA/CS (20%)/fullerenol (5%) composite supported on a UPM substrate with additional modification with five PSS/CS bilayers: 0.340 kg/(m^2^ h) permeation flux and 95.6 wt.% water content in the permeate. Thus, previous research demonstrated the simultaneous application of bulk and surface modifications as a prospective strategy for the development of high-performance membranes for pervaporation dehydration.

Along with the applications of various modification approaches, it is crucial to consider designing environmentally friendly membranes. In particular, conventional fossil-based polymers employed for membrane production can be easily replaced with biopolymers obtained from bacterial fermentation products, plants, or animal sources. Nevertheless, the use of such biopolymers in membrane technology requires further research and development, initially at a laboratory scale, in order to enable further expansion into industrial applications [46].

The aim of this work was to further improve membranes based on biopolymer sodium alginate (SA) modified with water-soluble fullerene derivatives (fullerenol and fullerene derivative with L-arginine) for pervaporation dehydration [47,48]. The novelty of this work is the application of various strategies to improve the transport properties of SA pervaporation membranes: (I) the adequate selection of a porous substrate for the creation of a supported SA membrane, (II) the deposition of nano-sized thin PEL layers using the Lbl method on the surface of supported SA membranes, and (III) the study of uncross-linked developed supported membranes based on SA and its related composites with fullerene derivatives. The transport properties of the prepared membranes were tested using the pervaporation dehydration of isopropanol. Two main points were investigated: (I) the impacts of commercial porous substrates made from polyacrylonitrile (PAN), regenerated cellulose (RC), and aromatic polysulfone amide (UPM) were investigated by scanning electron microscopy (SEM), atomic force microscopy (AFM), the standard porosimetry method, and water filtration, in order to study mass transfer in the pervaporation dehydration of isopropanol; and (II) the effects of the PEL pair and the number of PEL bilayers formed using Lbl deposition on the properties of the SA membranes were studied by SEM, AFM, and pervaporation, in order to assess the prospects of the application of supported membranes modified by PEL in industrial dehydration processes. The stability of top PEL nanolayers deposited by Lbl assembly on the surface of the PAN-supported SA membranes was studied by contact angle measurements, AFM, and SEM. We additionally tested the potential use of the designed surface-modified membranes in more realistic industrial conditions: (I) the separation of mixtures with a higher water content (up to 70 wt.%), (II) studying the membranes’ stability after the experiment, and (III) the dehydration of other organic solvents such as ethanol and tetrahydrofuran.

## 2. Materials and Methods

### 2.1. Materials

Sodium alginate, (SA, Jiangsu Benefit Ocean Technology Co. Ltd., Lianyungang, China) obtained from BIOPROD Ltd. (St. Petersburg, Russia), was used as a membrane material. Fullerene derivatives—polyhydroxylated fullerene (HF, fullerenol, C_60_(OH)_22–24_) and a fullerene derivative with L-arginine (AF, C_60_-Arg, (C_60_(C_6_H_13_N_4_O_2_)_8_H_8_))—were purchased from Fullerene Technologies (St. Petersburg, Russia) and used for the bulk modification of SA. Isopropanol (iPrOH), ethanol (EtOH), tetrahydrofuran (THF), and calcium chloride (CaCl_2_) obtained from Vekton (St. Petersburg, Russia) were used without additional purification. 

Commercial porous membranes from polyacrylonitrile (PAN, Institute “Leibniz-Institut für Polymerforschung Dresden”, Dresden, Germany), regenerated cellulose (RC, Institute of Physical Organic Chemistry of the National Academy of Sciences of Belarus, Minsk, Belarus), and aromatic polysulfone amide (UPM, “Vladipor”, Vladimir, Russia) were used as substrates for the preparation of supported membranes with a thin selective layer based on SA. For the surface modification of the supported SA membranes using the Lbl technique, poly(sodium 4-styrene sulfonate) (PSS, M_W_ ~70,000 Da, Sigma-Aldrich, St. Petersburg, Russia) and polyacrylic acid (PAA, M_W_ ~100,000 Da, Sigma-Aldrich, St. Petersburg, Russia) were used as polyanions; poly(allylamine hydrochloride) (PAH, M_W_~50,000 Da, Sigma-Aldrich, St. Petersburg, Russia) and chitosan (CS, with low molecular weight, Sigma-Aldrich, St. Petersburg, Russia) were used as polycations.

### 2.2. Membrane Preparation

#### 2.2.1. Supported Membranes

The SA solution and composites with fullerene derivatives were prepared according to the following procedure: C_60_(OH)_22–24_ or C_60_-Arg (0 and 5 wt.% with respect to the SA weight) were added to the 1 wt.% SA solution, prepared by dissolving the polymer in water with stirring for 4 h at 45 °C, by solution method (the addition of HF dispersion in water) or by solid-phase synthesis (ground polymer with AF powders), respectively [47,48]. After that, the SA solution and composites were subjected to ultrasonic treatment. For the preparation of supported membranes, the prepared solutions were cast onto the surface of porous substrates made of polyacrylonitrile (PAN), regenerated cellulose (RC), and aromatic polysulfone amide (UPM) in the following manner: the substrate was fixed on a steel hollow ring, the prepared solution was cast onto the substrate from the ring side, and the excess casting solution was removed. Afterwards, the prepared supported membranes were left to dry at room temperature for 24 hours to evaporate the solvent. The prepared membranes were used either without additional treatment or after chemical cross-linkage with calcium chloride. The cross-linkage of the supported membranes was carried out by immersing them in 1.25 wt.% CaCl_2_ aqueous solution for 10 minutes followed by rinsing with deionized water [49].

#### 2.2.2. Surface Modification with Layer-by-Layer (Lbl) Assembly

**Figure 1:**
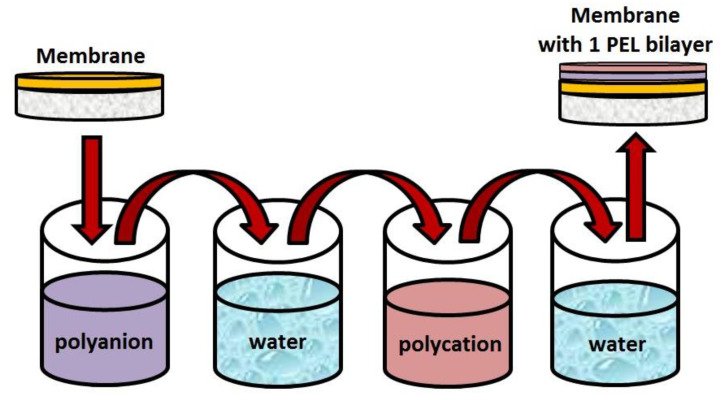
Layer-by-layer (Lbl) deposition scheme.

For the surface modification of the cross-linked supported membranes by Lbl assembly, the Xdip-MV1 robotic immersion coating system (“PROMENERGOLAB” Ltd., Moscow, Russia) was used. PSS, PAA, PAH, SA (10^−2^ mol/L), and CS (4.7 wt.% in 1 wt.% acetic acid solution) were used as PEL solutions. The membrane was attached to a Teflon plate with a selective layer oriented outward by fixing with silica gel around the circumference of the membrane to prevent the exposure of the porous substrate to PEL and water. The membrane was alternately immersed in PEL solutions for 10 minutes and washed with water between the immersions [27]. The polyanion solution of the PSS or PAA was deposited initially, since SA and polyanions may form intermolecular hydrogen bonds creating a stable polyanion layer on the membrane surface. The interaction between PSS (polyanion) and SA was confirmed by Fourier transform infrared (FTIR) spectroscopy by Sarwar et al. [50]. After the deposition of the polyanions, the membrane was washed with water several times. The next layer was applied with a polycation solution (CS, PAH, or SA), with subsequent rinsing in water, which completed the formation of a PEL bilayer on the membrane surface. The Lbl procedure is schematically illustrated in Figure 1. In this study, the deposition of five bilayers was demonstrated to be the optimal number of depositions of PEL, resulting in improved membrane properties. Previously, it was also shown that if the membrane contained one of the polyelectrolytes in bulk, then five PEL bilayers, containing the same polyelectrolyte, were sufficient for the uniform coverage of the entire membrane surface and led to significant changes in the transport properties [40]. 

The charge concentration *ρ_c_* of PEL pairs was calculated as the ratio of the number of ion pairs (in our case = 1) to carbon atoms per unit of the anionic and cationic PEL [51]: for PSS/CS this value was 1/(8 + 8) = 0.0625; for PSS/PAH, 1/(8 + 3) = 0.09; and for PSS/SA, 1/(12 + 8) = 0.05.

The designations and preparation conditions of the supported membranes are given in Table 1. Porous substrates for the supported membranes are indicated by a slash; concentrations of fullerene derivatives in the SA membranes are indicated as 5HF or 5AF after a dash; the number of PEL bilayers deposited on the membrane surface is indicated as 5Lbl through a slash; the cross-linking agent CaCl_2_ and PEL combination used for surface modification of the membrane are shown as abbreviations in the superscript.

### 2.3. Pervaporation

The performance of the supported membranes was evaluated by the pervaporation dehydration of isopropanol (12–70 wt.% water), ethanol (4–70 wt.% water), and THF (5.7–70 wt.% water) at ambient temperature (22 °C) using a steady-state regime cell [47]. The composition of the permeate and the feed was analyzed by gas chromatography using a Chromatek Crystal 5000.2 chromatograph (Chromatec, Nizhny Novgorod, Russia) with a “Hayesep R” column and a thermal conductivity detector. The transport properties (permeation flux, water content in the permeate, pervaporation separation index, separation factor, and component permeances) of the obtained membranes were calculated.

The permeation flux *J* (kg/(m^2^ h)) was calculated by the following equation [52]:

(1) $$J=\frac{W}{A\times t},$$

where *W* is the weight of the permeate (kg), *A* is the membrane effective area (m^2^), and *t* is the time of permeate collecting (h).

The separation factor (*β*) was calculated by the following equation [53]:

(2) β=yiyjxixj,

where *y_i_* and *y_j_* are the *i* and *j* component weight fractions in the permeate, *x_i_* and *x_j_* are *i* and *j* component weight fraction in the feed.

The pervaporation separation index, including the permeation flux and separation factor, was calculated by the following equation [54]:

(3) PSI=J·(β−1),

The component permeance was calculated by the following equation [53]:

(4) $$\frac{P}{l}=\frac{j_{i}}{p_{i_{f}}-p_{i_{p}}},$$

where *l* is the membrane thickness, $p_{i_{f}}$ and $p_{i_{p}}$ are the *i* component vapor pressures in the feed and the permeate, respectively, and *j_i_* is the partial flux of *i* component.

Each pervaporation measurement was carried out more than three times, and the average value was obtained for subsequent analysis. The mean accuracy for the transport parameters was as follows: ±0.5% for water content in the permeate and ±1% for the permeation flux.

### 2.4. Scanning Electron Microscopy (SEM)

The cross-sectional and surface structures of the porous substrates and supported membranes were analyzed with a Zeiss Merlin SEM (Carl Zeiss SMT, Oberhochen, Germany) at a low accelerating voltage (1 kV). The cross-section of the membrane was obtained by the immersion of the membrane in liquid nitrogen its breaking perpendicular to the surface.

### 2.5. Atomic Force Microscopy (AFM)

The surface topography of porous substrates and supported membranes was studied with an NT-MDT NTegra Maximus atomic force microscope (NT-MDT Spectrum Instruments, Moscow, Russia) in tapping mode and with standard silicon cantilevers (15 N·m^−1^ rigidity).

### 2.6. The Standard Porosimetry Method

The total porosimetry of the porous PAN, RC, and UPM substrates was measured by the standard porosimetry method using a Porosimeter 3.1 instrument (POROTECH Ltd., Ontario, Canada). The substrate samples were prepared in tablet form with 23 mm diameter; *n*-octane was used as a reference liquid.

### 2.7. Filtration Performance of Substrates

The water flux of porous substrates (PAN, RC, and UPM) was measured in a custom-made stirred dead-end filtration system at the transmembrane pressure of 1 bar and 22 °C; described in detail in a previous study [27]. The permeation flux (*J*) of water was calculated by the following equation [55]:

(5) $$J=\frac{V}{A\times t},$$

where *V* is the permeate volume (L), *A* is the membrane effective area (m^2^), and *t* is the permeation time (h).

### 2.8. Contact Angle Measurement

To assess the surface hydrophilicity of the supported membranes, contact angles of water were investigated using a Goniometer LK-1 device (NPK Open Science Ltd., Krasnogorsk, Russia) by the sessile drop method. “DropShape” software was used for the analysis of the obtained contact angle data. The contact angle of the water was measured only on the selective top layer of the supported membranes, which contacted the feed.

## 3. Results

This part is divided into several sections.

Section 3.1, “The development of the supported SA membranes” is dedicated to the selection of a porous substrate for the creation of supported membranes with a thin selective SA-based layer and the testing of the developed supported membrane with the pervaporation dehydration of isopropanol (Section 3.1.1), as well as the investigation of the substrates by various techniques (Section 3.1.2) to define the component mass transfer in pervaporation through the supported SA-based membranes. 

In Section 3.2, “Surface modification of the supported SA and SA/fullerene derivative membranes by Lbl deposition of PEL”, the application of Lbl assembly for PEL deposition on developed PAN-supported membranes based on SA and SA-fullerene derivative (5%) composites is presented. In Section 3.2.1, the effect of PEL combinations and a number of PEL bilayers on the transport properties of the SA membrane in the pervaporation dehydration of isopropanol is studied. In Section 3.2.2, the further improvement of surface-modified membrane properties was achieved by the introduction of fullerene derivatives (HF and AF) into the SA matrix. Additionally, to study the membrane effectiveness in other pervaporation dehydration processes, the developed membrane, modified with HF and five PSS/SA bilayers, was tested via the pervaporation dehydration of ethanol and tetrahydrofuran in a wide concentration range. 

The last Section 3.3, “Comparison of the performance of the membranes with PEL layers with membranes described in the literature”, is devoted to the comparison of pervaporation performance of the developed PAN-supported SA-5HF membrane with five PSS/SA bilayers, with membranes described in the literature under close experimental conditions.

### 3.1. The Development of the Supported SA Membranes

#### 3.1.1. Transport Properties of the Supported SA Membranes

To increase the SA membrane efficiency for industrial application, a selective thin SA layer was deposited on various commercial porous substrates such as PAN, UPM, and RC to develop supported membranes. The transport properties of the prepared untreated supported SA/PAN, SA/UPM, and SA/RC membranes were tested in the pervaporation separation of an azeotropic water/isopropanol (12/88 wt.%) mixture at 22 °C (Table 2). The transport properties of the dense SA membrane (~25 µm thickness) are also demonstrated in Table 2 for comparison with the supported membranes [47].

The data in Table 2 demonstrate that the deposition of a thin selective SA layer on the commercial porous substrates UPM, RC, and PAN increases permeation flux by 15% (for SA/UPM), 32% (for SA/RC), and 76% (for SA/PAN), respectively, compared to the dense SA membrane. Additionally, the decrease of selective properties for the supported SA/UPM and SA/RC membranes was observed: water content in the permeate decreased to 98.7 and 99.4 wt.%, and the separation factor decreased to 536 and 1215, respectively. The highest value of PSI, a parameter used to characterize the efficiency of pervaporation separation, was exhibited by the developed supported SA/PAN membrane, indicating its efficiency and high-performance in the pervaporation separation of an azeotropic water/isopropanol mixture. Thus, the type of substrate polymer, its surface characteristics, and the porosity of the substrate have a significant effect on the transport properties of the developed supported membrane [27,56,57,58]. It is worth clarifying that the sieve mechanism is not applicable for the explanation of transport properties of supported membranes [59], since, in our case, the separated mixture first comes into contact with the top thin dense SA-based layer (without pores). The separation of components through this layer occurs due to the free volume between the polymer chains according to the “solubility-diffusion” mechanism [60]. The substrate of the supported membrane functions as mechanical support for the pervaporation-active top selective dense layer based on SA and contributes to the separation of components to a much lesser extent than the non-porous selective top layer. The characteristics of the applied substrates are considered in detail below in Section 3.1.2, “The investigation of the substrates”.

**Figure 2:**
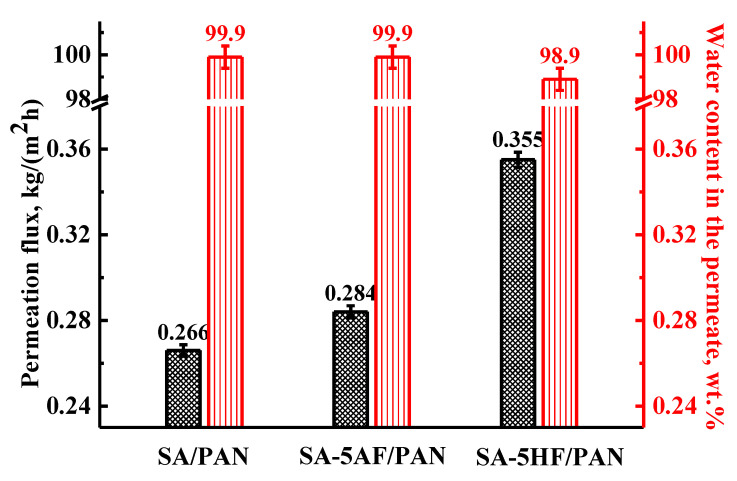
Permeation flux and water content in the permeate for the untreated PAN-supported membranes based on SA, SA-AF (5%), and SA-HF(5%) composites in the pervaporation separation of an azeotropic water/isopropanol (12/88 wt.%) mixture at 22 °C.

To increase further the performance of the PAN-supported SA membrane, mixed matrix membranes were developed by the introduction of 5 wt.% fullerenol (HF) and fullerene derivative with L-arginine (AF) into the thin, selective SA layer. The transport properties of the untreated (uncross-linked) PAN-supported membrane based on SA and its SA-HF (5%) and SA-AF (5%) composites were tested also in the pervaporation separation of an azeotropic water/isopropanol (12/88 wt.%) mixture. The results are presented in Figure 2.

It was demonstrated that the bulk modification of the untreated PAN-supported SA membrane with AF and HF nanoparticles (SA-5AF/PAN and SA-5HF/PAN) increased permeation flux by 7 and 33%, respectively, for the pervaporation separation of an azeotropic water/isopropanol (12/88 wt.%) mixture. At the same time, a slight decrease in the selectivity (98.9 wt.% water content in the permeate) was observed for the supported SA-5HF/PAN membrane, while the SA/PAN and SA-5AF/PAN membranes showed 99.9 wt.% water content in the permeate. The increased permeability of the PAN-supported membrane with HF compared to others was related to the specific interaction of HF with SA, and the presence of a large number of polar hydroxyl groups in HF. In our previous work [47], for the untreated SA-5HF membrane, the formation of hydrogen bonds between HF and SA was confirmed by FTIR spectroscopy. Binding between HF and SA significantly facilitated the maintenance of a high level of membrane selectivity with a strong increase in permeability [47]. Additionally, the modification of the SA matrix by HF to a greater extent enhanced the roughness of the inner membrane structure, compared to the membrane modified by AF. This increased the permeability of the HF-modified membrane [47,48]. SEM data previously confirmed this increased roughness.

**Figure 3:**
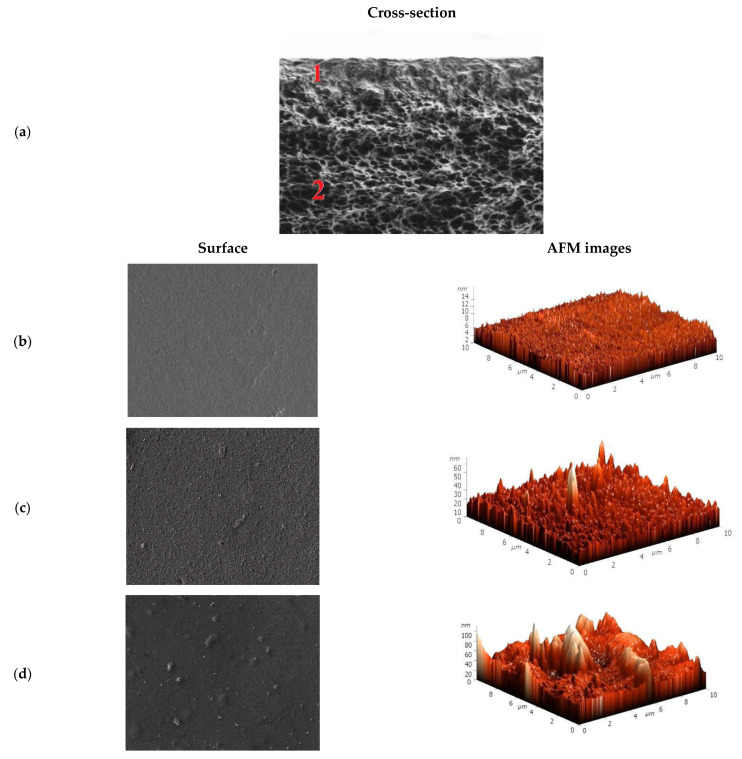
(**a**) The cross-sectional micrograph of the SA/PAN membrane, surface SEM micrographs, and AFM images of the supported (**b**) SA/PAN, (**c**) SA-5HF/PAN, and (**d**) SA-5AF/PAN membranes.

To explain the mass transfer of components in pervaporation, the inner and surface morphology of the PAN-supported membranes based on SA and its composites with HF and AF were investigated by SEM and AFM. The cross-sectional micrograph is presented only for the SA/PAN membrane (Figure 3a) since, for the modified membranes (SA-5HF/PAN and SA-5AF/PAN), they were identical to the membrane based on the pristine polymer. The surface SEM micrographs and AFM images with a scan size of 10 × 10 μm for the PAN-supported membranes are also presented in Figure 3.

On the cross-sectional SEM micrograph of the SA/PAN membrane, there are two distinct areas (Figure 3a): (1) the top area of the selective dense layer based on SA with a thickness of ~1 µm, and (2) the area of the porous PAN support. The surface of the SA/PAN membrane had a relatively smooth and uniform structure (Figure 3b), while the modification of this membrane by fullerene derivatives changed the membrane surface structure. The introduction of HF into the SA matrix (SA-5HF/PAN membrane) resulted in a rougher membrane surface (Figure 3c) compared to the unmodified SA/PAN membrane, while for the SA-5AF/PAN membrane a large number of nanoparticles were observed on the surface (Figure 3d). The presence of more nanoparticles significantly affected the surface roughness of the membrane. The described results are in agreement with the data obtained from the AFM images (Figure 3b–d) presented in Table 3—the roughness parameters of the PAN-supported membranes (average (Ra) and root-mean-squared (Rq) surface roughness).

The data in Table 3 demonstrate the increase of surface roughness of the membranes modified by HF and AF compared to the SA/PAN membrane. The highest surface roughness parameters (Ra = 10.5 nm, Rq = 14.9 nm) were observed for the SA-5AF/PAN membrane, due to the emergence of carbon nanoparticles on the membrane surface (confirmed by SEM data, Figure 3d). For the SA-5HF/PAN membrane, the Ra and Rq values increased by 2.8 and 4 nm compared to the SA/PAN membrane, which was also related to a change in the surface morphology of the membrane during the modification process (SEM data, Figure 3c). An increase in surface roughness contributed to the formation of a larger number of sorption centers on the membrane surface, causing the permeability of the membranes modified by AF and HF to be improved compared to the SA/PAN membrane in the pervaporation separation of an azeotropic water/isopropanol (12/88 wt.%) mixture (Figure 2). Further improvement of the transport properties of the PAN-supported membranes based on SA and its composites with HF and AF was achieved by surface modification, through the application of polyelectrolytes on the membrane surface by Lbl method (Section 3.2. “Surface modification of the supported SA and SA/fullerene derivative membranes by Lbl deposition of PEL”).

#### 3.1.2. The Investigation of the Substrates

**Figure 4:**
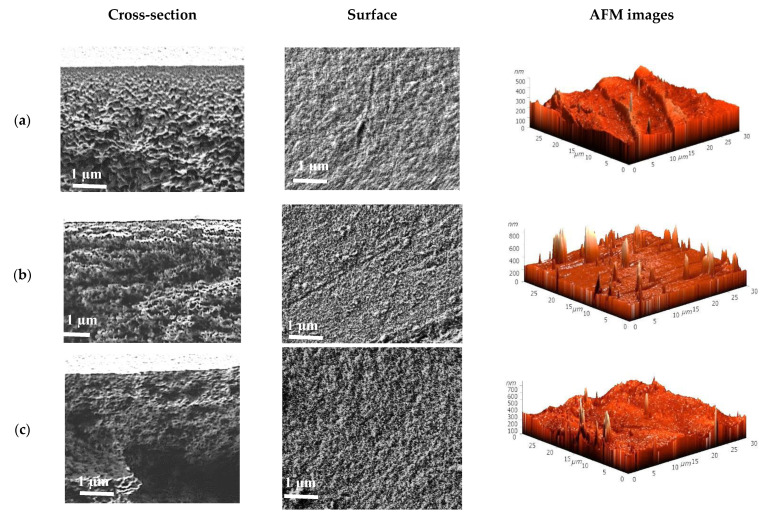
The cross-sectional and surface SEM micrographs and AFM images of the porous (**a**) UPM, (**b**) RC, and (**c**) PAN substrates.

To understand the mass transfer mechanism in pervaporation using the supported SA membranes, the characteristics of porous PAN, UPM, and RC substrates were studied by SEM, AFM, the standard porosimetry method, and water ultrafiltration experiments. The inner morphology and surface topography of the porous substrates were investigated by SEM and AFM. The cross-sectional and surface SEM micrographs and AFM images with a scan size of 30 × 30 μm for the substrates are presented in Figure 4.

The presented cross-sectional SEM micrographs (Figure 4) demonstrate that the bulk porosity of the PAN substrate is much higher than that of the UPM and RC substrates, due to the presence of large macrovoids. The macrovoids cause reduced resistance during the transfer of components through the membrane, resulting in increased permeation flux in the SA/PAN membrane (Table 2). It is also worth noting that the cross-sectional structure of all substrates was uneven, which is attributed to the viscosity of the casting polymer solution and the preparation phase inversion method [61,62]. The UPM substrate had a compact skin top layer, where the thickest transition from a dense structure with small pores (a top layer of the substrate, on which dense SA layer was deposited) to a porous structure with large pores in the cross-section, was observed. At the same time, for the PAN substrate this area was the thinnest, which could have affected the increased permeation flux of the SA/PAN membrane. The surfaces of the UPM and RC substrates are comparatively equally rough on the SEM micrographs, while the PAN substrate is characterized by a strongest surface roughness with the presence of a great number of small pores on the surface. Based on the AFM images (Figure 4), the roughness parameters of the substrate surfaces (average (Ra) and root-mean-squared (Rq) surface roughness) were calculated (Table 4). Additionally, to assess the substrate performance, total porosity and water permeability through the substrates were studied (Table 4).

It was demonstrated that the PAN substrate had the roughest surface (the highest values of Ra and Rq) compared to the UPM and RC substrates. These data are also in agreement with the surface SEM micrographs of the substrates (Figure 4). This implies that the PAN substrate has the largest effective surface in contact with the SA layer, which is responsible for the effective coating of a thin selective layer on the substrate. Additionally, it was demonstrated that the total porosity of the PAN substrate was the highest, which might be related to the presence of a large network pores on the top layer and the sublayer, as well as large macrovoids in the bulk of the substrate [63]. However, the value of the total porosity of UPM (95.0%) was comparable to PAN (96.5%). This finding may be explained by the great number of small pores in UPM and their sizes, shapes, number, and distribution nature in the volume of the substrate. To study the surface porous layer of the substrate, the water permeability of substrates was measured by ultrafiltration. The permeability showed the following increasing trend: UPM < RC < PAN. The highest water permeability of the PAN substrate was attributed to increased porosity and surface roughness, the largest pore size of the skin top layer, and the hydrophilicity of the material [61]. Thus, the PAN substrate significantly improved transport properties of the developed supported membrane with a thin selective layer based on SA.

### 3.2. Surface Modification of the Supported SA and SA/Fullerene Derivative Membranes by Lbl Deposition of PEL

#### 3.2.1. The Investigation of Membranes Based on Parent SA

**Figure 5:**
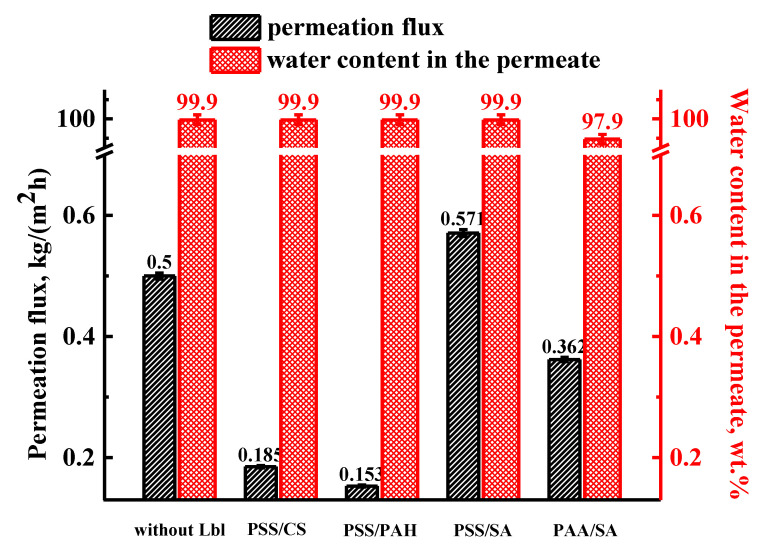
The transport parameters (permeation flux and water content in the permeate) of the cross-linked PAN-supported membranes based on SA without and with surface modification with five bilayers of different polyelectrolyte combinations (PSS/CS, PSS/PAH, PSS/SA, and PAA/SA) by Lbl assembly in the pervaporation separation of an azeotropic water/isopropanol (12/88 wt.%) mixture at 22 °C.

The Lbl technique of PEL deposition was used to improve the performance of the developed PAN-supported SA membranes. An improvement in the transport properties of the membranes was expected due to the more hydrophilic membrane surface containing PEL, inducing the increase of the charge density, which would facilitate the transfer of water molecules through the membrane [41]. To increase the membranes’ stability in water for further application in the pervaporation dehydration of mixtures with high water content, the developed PAN-supported membranes were subjected to cross-linking with the most commonly used cross-linking agent, calcium chloride [48], before surface Lbl modification. In order to select the optimal conditions of Lbl modification, the cross-linked supported membrane based on unmodified SA (SA/PAN^CaCl2^) was first coated with five bilayers of various PEL combinations, such as PSS/CS, PSS/PAH, PSS/SA, and PAA/SA. The transport properties of these surface-modified membranes (Figure 5) were investigated in the pervaporation separation of an azeotropic water/isopropanol (12/88 wt.%) mixture. The transport properties of the SA/PAN^CaCl2^ membrane without Lbl modification are presented in Figure 5 for comparison with the modified membranes.

It was demonstrated that Lbl modification with five bilayers of PSS/CS and PSS/PAH pairs decreased the permeation flux to 0.185 and 0.153 kg/(m^2^ h), respectively, compared to the SA/PAN^CaCl2^ membrane, maintaining 99.9 wt.% water in the permeate (Figure 5). The coating with five bilayers of PSS/SA on the SA/PAN^CaCl2^ membrane increased permeation flux by 14%, maintaining 99.9 wt.% water in the permeate. The increase of membrane permeability of the SA/PAN^CaCl2^-5Lbl^PSS/SA^ membrane could be attributed to reduced electrostatic cross-linking of this PEL pair, the formation of small hydrophilic mashes in the PEL layers, and the increased surface hydrophilicity of the modified membrane, which resulted in improved water penetration through the membrane in comparison to isopropanol [41,42]. Besides, the application of SA, which also constituted the membrane matrix, for Lbl modification, promoted improved membrane transport parameters due to extrinsic and intrinsic charge overcompensation and the competitive pairing of ions [39]. The same effect has also been demonstrated earlier [40]. The decreased permeation fluxes of the SA/PAN^CaCl2^ membranes with the deposition of five bilayers of the PSS/CS and PSS/PAH pairs may be related to the higher charge concentration of these PEL combinations (0.09 for PSS/PAH and 0.0625 for PSS/CS) compared to PSS/SA (0.05). Another reason may be a much denser PEL electrostatic cross-linking [39,51], which hampers and slowed down the mass transfer of substances through the membranes. The replacement of the strong PSS polyanion by a weak PAA paired with SA also decreased the membrane transport parameters of the surface-modified membrane compared to the SA/PAN^CaCl2^ and SA/PAN^CaCl2^-5Lbl^PSS/SA^ membranes. There was a decrease in permeation flux to 0.362 kg/(m^2^ h) with 97.9 wt.% water in the permeate (Figure 5). This effect may be explained as follows: combining a strong (fully charged) PEL (SA) with a weak PEL (PAA) with a variable charged state led to the reduced charge density of PAA in the multilayer, causing the significantly increased thickness of the PEL bilayer [64]. It affects the formation of a denser and longer diffusion path for separating substances through the membrane, reducing the transport characteristics of the membrane. 

To investigate the effect of the bilayer number on the transport properties of the surface-modified SA membranes, we developed membranes with three and ten bilayers of PSS/CS, PSS/PAH, and PSS/SA combinations. The coating of three bilayers of these PEL on the SA/PAN^CaCl2^ membrane did not change transport characteristics in the pervaporation separation of an azeotropic water/isopropanol mixture, compared to the membrane unmodified by PEL (SA/PAN^CaCl2^); the results for the unmodified by PEL membrane are also presented in Figure 5 for comparison. This indicated that the deposition of this number of PEL bilayers was not sufficient to form a uniform top PEL layer without defects on the membrane surface [40]. The increase of the PEL bilayer number to ten enhanced permeation fluxes to 0.286 and 0.214 kg/(m^2^ h) with 99.9 wt.% water in the permeate for the PSS/CS and PSS/PAH-modified SA/PAN^CaCl2^ membranes, respectively. The coating with ten bilayers of PSS/SA resulted in a decrease in the permeation flux to 0.516 kg/(m^2^ h), maintaining 99.9 wt.% water in the permeate in the pervaporation separation of an azeotropic water/isopropanol mixture compared to the SA/PAN^CaCl2^-5Lbl^PSS/SA^ membrane.

Thus, based on the pervaporation data, the optimal conditions of the surface modification of the SA/PAN^CaCl2^ membrane by Lbl technique was the deposition of five PSS/SA bilayers, which resulted in an improved membrane performance (increased permeability with high selectivity with respect to water). The further improvement of membrane properties was carried out by a combination of bulk (the introduction of fullerene derivatives into the SA matrix) and surface (coating with optimal five PSS/SA bilayers on the membrane surface) modifications.

#### 3.2.2. The Investigation of Membranes Based on SA/Fullerene Derivative Composites

**Figure 6:**
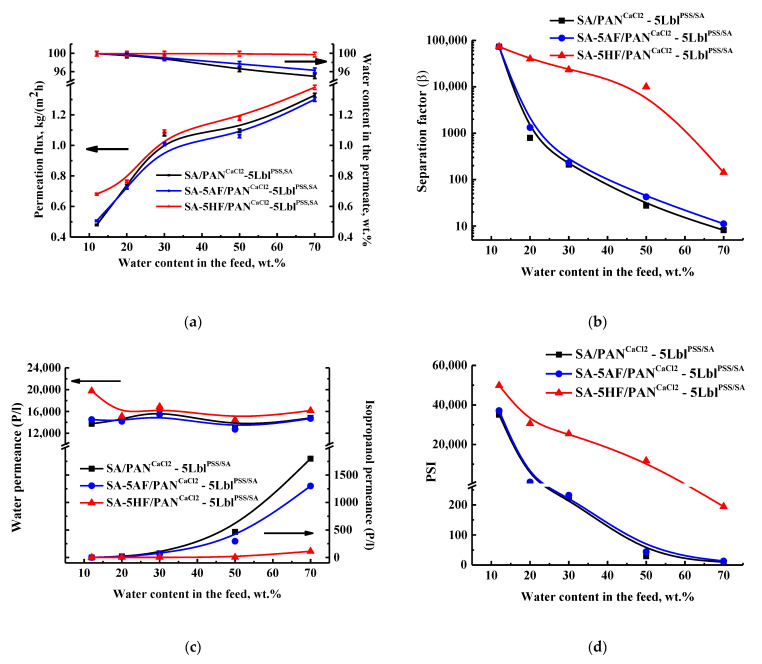
The dependence of (**a**) the water content in the permeate and the permeation flux, (**b**) the separation factor, (**c**) the component permeances, and (**d**) the PSI on the water content in the feed for the cross-linked PAN-supported membranes based on SA, SA-AF (5%), and SA-HF (5%) composites with five PSS/SA bilayers in the pervaporation dehydration of isopropanol (12–70 wt.% water) at 22 °C.

In industry, output water/organic solvent mixtures often have a high water content. Therefore, the transport properties (permeation flux, component permeances, water content in the permeate, separation factor, and PSI) of the cross-linked PAN-supported SA/fullerene derivative (5 wt.% HF or AF) membranes with the deposited five PSS/SA bilayers were studied in the pervaporation dehydration of isopropanol in a wide concentration range (12–70 wt.% water, Figure 6) to assess their prospective industrial application. The transport properties of the SA/PAN^CaCl2^-5Lbl^PSS/SA^ membrane are also shown for comparison with the modified membranes.

It was demonstrated that the modification of the SA/PAN^CaCl2^ membrane with 5 wt.% AF and HF with the deposition of five PSS/SA bilayers improved its selective properties (over 96.3 and 99.7 wt.% water in the permeate, respectively, Figure 6a). A not significant decrease in the permeation flux for the SA-5AF/PAN^CaCl2^-5Lbl^PSS/SA^ by 2–6% compared to the SA/PAN^CaCl2^-5Lbl^PSS/SA^ membrane was observed. The SA-5HF/PAN^CaCl2^-5Lbl^PSS/SA^ membrane had the highest values of the permeation flux (0.68–1.38 kg/(m^2^ h), Figure 6a). This effect is related to a large number of polar hydroxyl groups in HF capable of considerably reducing the effect of the cross-linking agent calcium chloride compared to AF [47,48]. Thus, the introduction of HF increased membrane permeability. At the same time, HF acts as a modifier and, due to a rough membrane surface, provides better adhesion for PEL layers and the highly selective separation of the components in a pervaporation process [47]. The calculated separation factor based on the obtained data regarding water content in the permeate also confirmed the most selective properties for the SA-5HF/PAN^CaCl2^-5Lbl^PSS/SA^ membrane (Figure 6b). The calculated component permeances also demonstrate that the SA-5HF/PAN^CaCl2^-5Lbl^PSS/SA^ membrane is characterized by the highest water penetration and the lowest isopropanol penetration (Figure 6c), which contributes to the best overall membrane selective properties (Figure 6a,b). To evaluate the overall pervaporation performance, considering membrane permeability and selectivity, PSI was also calculated [54,65]. PSI values demonstrated the highest effectiveness of the SA-5HF/PAN^CaCl2^-5Lbl^PSS/SA^ membrane (Figure 6d). 

**Figure 7:**
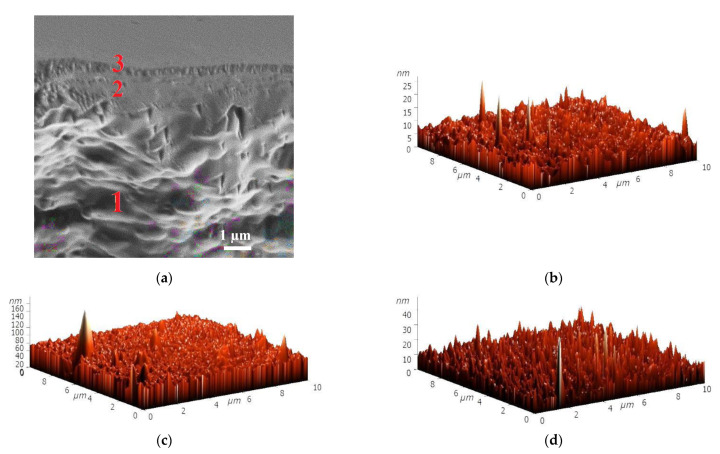
(**a**) A cross-sectional SEM micrograph of the SA-5HF/PAN^CaCl2^-5Lbl^PSS/SA^ membrane and surface AFM images for the (**b**) SA/PAN^CaCl2^-5Lbl^PSS/SA^, (**c**) SA-5AF/PAN^CaCl2^-5Lbl^PSS/SA^, and (**d**) SA-5HF/PAN^CaCl2^-5Lbl^PSS/SA^ membranes.

The inner morphology and surface topography of the membranes were studied by SEM and AFM to demonstrate the effect of Lbl modification. A cross-sectional SEM micrograph of the SA-5HF/PAN^CaCl2^-5Lbl^PSS/SA^ membrane and surface AFM images with a scan size of 10 × 10 μm for the surface-modified membranes are presented in Figure 7. The cross-sectional SEM micrographs of all membranes were identical.

On the cross-sectional SEM micrograph of the SA-5HF/PAN^CaCl2^-5Lbl^PSS/SA^ membrane, there are three distinct areas (Figure 7a): (1) the area of the porous PAN support, (2) the area of the selective layer based on the SA-HF (5%) composite with a thickness of 1.0 ± 0.3 µm, and (3) the area of the thin PEL layer of PSS/SA with a thickness of 90 ± 10 nm. The surface roughness parameters in terms of average (Ra) and root-mean-squared (Rq) roughness, calculated based on the AFM images (Figure 7b–d), are presented in Table 5.

It was demonstrated that the cross-linking and deposition of the PSS/SA layers for the SA/PAN membrane led to a slight increase of surface roughness (0.7 nm of Ra, 0.9 nm of Rq) compared to the SA/PAN membrane (Ra = 0.7 nm, Rq = 0.9 nm, Table 3). For the modified SA-5AF/PAN^CaCl2^-5Lbl^PSS/SA^ and SA-5HF/PAN^CaCl2^-5Lbl^PSS/SA^ membranes, a decrease in surface roughness parameters is observed in comparison with the untreated SA-5AF/PAN and SA-5HF/PAN membranes (Table 3), due to the formation of a 90 nm-thick PEL layer covering all significant irregularities and nanoparticles on the membrane surface. The SA-5AF/PAN^CaCl2^-5Lbl^PSS/SA^ membrane had the roughest surface compared to the SA/PAN^CaCl2^-5Lbl^PSS/SA^ and SA-5HF/PAN^CaCl2^-5Lbl^PSS/SA^ membranes. The same trend was also observed for the untreated PAN-supported membranes (Table 3). This could be related to the fact that AF nanoparticles are present on the SA membrane surface (confirmed by SEM data, Figure 3) and the subsequent application of five PSS/SA bilayers also preserved this rough surface structure [48]. However, the presence of fullerenol on the SA-5HF/PAN^CaCl2^-5Lbl^PSS/SA^ membrane only slightly increased the surface roughness compared to the SA/PAN^CaCl2^-5Lbl^PSS/SA^ membrane. Notably, the difference in the surface roughness of the Lbl-modified membranes did not exceed 10 nm, which is highly unlikely to significantly affect the changes in the transport parameters. However, the deposition of the PSS/SA bilayers on the membrane surface enhanced the affinity of the dense selective layer for water due to the surface charge and the formation of hydrophilic mashes related to electrostatic PEL interactions, whereas the dense selective layer based on SA and its composites was responsible for a deeper separation of the mixture.

**Figure 8:**
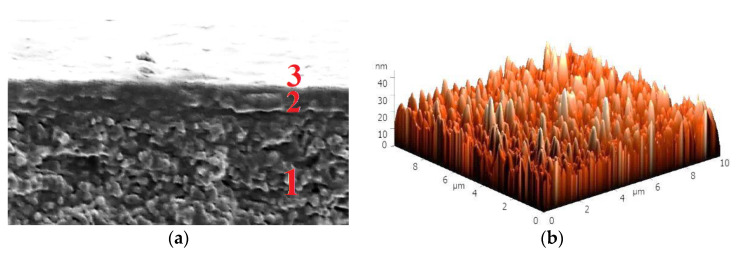
(**a**) A cross-sectional SEM micrograph and (**b**) a surface AFM image of the SA-5HF/PAN^CaCl2^-5Lbl^PSS/SA^ membrane after the pervaporation dehydration of isopropanol (12–70 wt.% water).

Thus, based on its transport properties in the pervaporation dehydration of isopropanol (Figure 6), the SA-5HF/PAN^CaCl2^-5Lbl^PSS/SA^ membrane exhibited the optimal performance. The stability of the PEL layers of this membrane was studied by SEM and AFM after the pervaporation experiment, as well as the contact angle of water measurements before and after the pervaporation experiment. A SEM cross-sectional micrograph and a surface AFM image with a scan size of 10 × 10 μm after the pervaporation are presented in Figure 8.

In the cross-sectional SEM micrograph of the SA-5HF/PAN^CaCl2^-5Lbl^PSS/SA^ membrane, it was demonstrated that there were three areas with an unchanged layer thickness, which confirmed the indelibility and preservation of the PEL layer on the membrane surface without defects after the pervaporation (Figure 8a). The surface roughness parameters (Ra and Rq) of the SA-5HF/PAN^CaCl2^-5Lbl^PSS/SA^ membrane after the pervaporation dehydration of isopropanol were calculated based on the AFM image (Figure 8b). These were equal to 3.8 nm and 4.9 nm, respectively, which was not significantly higher compared to the surface parameters of the SA-5HF/PAN^CaCl2^-5Lbl^PSS/SA^ membrane before the pervaporation (at 1.4 nm for Ra and at 1.7 nm for Rq, Table 5). The retention of the surface roughness of the SA-5HF/PAN^CaCl2^-5Lbl^PSS/SA^ membrane after the pervaporation at the same level in comparison with the membrane surface roughness before pervaporation (Table 5) also indicates the stability of the PEL layers. The contact angles of water for the SA-5HF/PAN^CaCl2^-5Lbl^PSS/SA^ membrane before and after the pervaporation experiment were very close in value: 48 ± 5° before and 46 ± 6° after the pervaporation experiment. Thus, SEM, AFM, and contact angle data confirmed that the surface of the SA-5HF/PAN^CaCl2^-5Lbl^PSS/SA^ membrane did not deteriorate, which indicated that the PEL layers were stable and did not wash off during the separation process. Thus, the membranes with improved transport characteristics are promising tools for utilization in industrial dehydration in the future.

**Figure 9:**
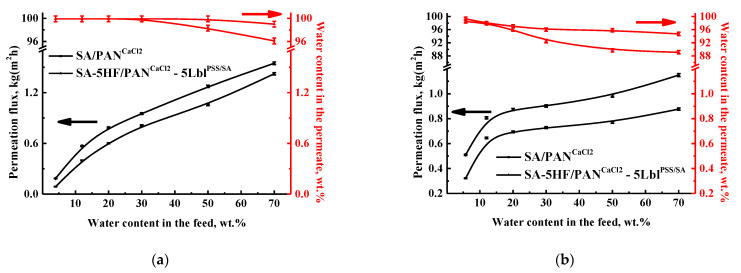
The dependence of the permeation flux and water content in the permeate on the water content in the feed for the SA-5HF/PAN^CaCl2^-5Lbl^PSS/SA^ and SA/PAN^CaCl2^ membranes in the pervaporation dehydration of (**a**) ethanol (4–70 wt.% water) and (**b**) THF (5.7–70 wt.% water) at 22 °C.

To confirm the effectiveness of the developed SA-5HF/PAN^CaCl2^-5Lbl^PSS/SA^ membrane in industrial application, this membrane was also evaluated in the pervaporation dehydration of other solvents, namely ethanol (EtOH) and tetrahydrofuran (THF). The use of traditional methods for the separation of water/EtOH and water/THF systems is impractical and energy-intensive, since it requires the use of an intermediate agent to facilitate the separation. In particular, both systems form azeotropic mixtures, adding the need for an additional purification stage of the target product [66,67]. In the current study, the pervaporation separation of EtOH/water and THF/water mixtures was carried out in a wide concentration range (4–70 wt.% and 5.7–70 wt.% water, respectively), including in the azeotropic mixtures (4.3/95.7 wt.% water/EtOH and 5.7/94.3 wt.% water/THF [68,69]). The transport properties of the SA-5HF/PAN^CaCl2^-5Lbl^PSS/SA^ membrane and the SA/PAN^CaCl2^ without Lbl modification are presented in Figure 9 for comparison.

It was demonstrated that the permeability of the SA-5HF/PAN^CaCl2^-5Lbl^PSS/SA^ membrane increased by 10–111% and 25–58% in the pervaporation dehydration of EtOH and THF, respectively, compared to the SA/PAN^CaCl2^ membrane without Lbl modification. Additionally, the water content in the permeate in the pervaporation dehydration of EtOH for the modified SA-5HF/PAN^CaCl2^-5Lbl^PSS/SA^ membrane was higher (over 99.0 wt.% water) compared to the SA/PAN^CaCl2^ membrane (over 94.7 wt.% water, Figure 9a). For the pervaporation dehydration of THF, we observed a not significant decrease in selective properties (89–98.5 wt.% water in the permeate) for the modified membrane compared to the SA/PAN^CaCl2^ membrane (94–99.3 wt.% water, Figure 9b).

To explain the obtained dependences of the dehydration of alcohols (ethanol and isopropanol) and THF, the contact angles of water, isopropanol, ethanol, and THF for the SA-5HF/PAN^CaCl2^-5Lbl^PSS/SA^ and SA/PAN^CaCl2^ membranes were measured. It was demonstrated that for the SA-5HF/PAN^CaCl2^-5Lbl^PSS/SA^ membrane (modified with 5% HF and five PSS/SA bilayers) the contact angle of water increased to 48° compared to the unmodified SA/PAN^CaCl2^ membrane (34°), which indicated the hydrophobization of the membrane’s surface. The contact angles of less polar components such as ethanol, isopropanol, and THF for both membranes were impossible to measure. The membranes exhibited a high affinity and complete surface wettability in these solvents that was in agreement with surface hydrophobization, confirmed by the contact angles of water. The obtained contact angle results were in agreement with the transport properties of the membranes in the pervaporation dehydration of ethanol, isopropanol, and THF, where the permeation flux for the PEL-containing membrane (SA-5HF/PAN^CaCl2^-5Lbl^PSS/SA^) was higher as compared to the unmodified membrane (SA/PAN^CaCl2^). The permeation flux in pervaporation dehydration (12 wt.% water) has a similar tendency to increase for both membranes in the following order: ethanol < isopropanol < THF. Such a pattern is associated with the differences in polarity of these solvents, causing the higher sorption of organic components on the membrane and facilitating mass transfer. It should be noted that the decrease of water content in the permeate for the SA-5HF/PAN^CaCl2^-5Lbl^PSS/SA^ membrane as compared to the SA/PAN^CaCl2^ membrane was found only in the dehydration of THF (Figure 9b). It may be explained by the lowest polarity of THF compared to alcohols (isopropanol and ethanol). Therefore, THF induces higher swelling of the PEL layer of the modified membrane. That leads to the co-penetration of THF and water through the membrane, resulting in a decrease in the selectivity of the SA-5HF/PAN^CaCl2^-5Lbl^PSS/SA^ membrane (Figure 9b).

To conclude, the developed SA-5HF/PAN^CaCl2^-5Lbl^PSS/SA^ membrane, modified by 5 wt.% HF and coated with five PSS/SA bilayers, showed improved transport characteristics in pervaporation dehydration not only for isopropanol (Figure 6) but also in the case of EtOH and THF (Figure 9), which demonstrated the membrane’s promise for industrial use in dehydration processes.

### 3.3. Comparison of the Performance of the Membranes with PEL Layers with Membranes Described in the Literature

The transport properties comparison of the SA-5HF/PAN^CaCl2^-5Lbl^PSS/SA^ membrane to the supported membranes with the surface modification (with the same or similar PEL layers) described in the literature for the pervaporation dehydration of isopropanol is summarized in Table 6.

It was demonstrated that the developed SA-5HF/PAN^CaCl2^-5Lbl^PSS/SA^ membrane had improved transport properties (high permeation flux of 0.765 kg/(m^2^ h) and the highest selectivity level—over 99.9 wt.% water in the permeate) in the pervaporation dehydration of isopropanol (20 wt.% water) compared to the supported membranes with surface modification by the Lbl technique described in the literature [27,40,41,70]. Additionally, it is worth noting that the membranes developed in our previously published article [70], HEC/SA-fullerenol (5%)-PAN^CaCl2^ membranes modified with five bilayers of PSS/PAH and PSS/SA exceeded the performance of the SA-5HF/PAN^CaCl2^-5Lbl^PSS/SA^ membrane obtained in this article. It was demonstrated that the addition of HEC to SA increased the permeability of the membrane (by 28% for the HEC/SA-fullerenol (5%)/PAN^CaCl2^-Lbl^PSS,PAH^ membrane and by 13% for the HEC/SA-fullerenol (5%)/PAN^CaCl2^-Lbl^PSS,SA^ membrane), but with a significant decrease of selective properties (decreased the separation factor to 52 and 156, respectively) compared to the developed SA-5HF/PAN^CaCl2^-5Lbl^PSS/SA^ membrane.

The comparison of the transport properties of the SA-5HF/PAN^CaCl2^-5Lbl^PSS/SA^ membrane to the dense and supported SA-based membranes and the commercial PERVAP^TM^ 1201 membrane (Sulzer Chemtech) described in the literature for the pervaporation dehydration of isopropanol under close experimental conditions is summarized in Table 7.

It was demonstrated that the developed SA-5HF/PAN^CaCl2^-5Lbl^PSS/SA^ membrane has good transport properties in the pervaporation dehydration of isopropanol (12 and 30 wt.% water) compared to the SA-based membranes described in the literature. There are several types of membranes, which have higher permeation flux in the pervaporation separation of a water/isopropanol (30/70 wt.%) mixture compared to the developed SA-5HF/PAN^CaCl2^-5Lbl^PSS/SA^ membrane. However, the SA-5HF/PAN^CaCl2^-5Lbl^PSS/SA^ membrane has improved selective properties (separation factor of 23,331) in the pervaporation separation of a water/isopropanol (30/70 wt.%) mixture. It is also worth noting that the permeation flux of the developed membrane is 24 times higher compared to the commercial dehydration PERVAP^TM^ 1201 membrane in the pervaporation dehydration of isopropanol (12 wt.% water) at 22 °C.

The comparison of the transport properties of the SA-5HF/PAN^CaCl2^-5Lbl^PSS/SA^ membrane to the other types of membranes described in the literature for the pervaporation dehydration of ethanol under close experimental conditions is summarized in Table 8.

Based on the data of Table 8, the developed SA-5HF/PAN^CaCl2^-5Lbl^PSS/SA^ membrane also has improved transport properties (high permeation flux of 0.185–0.785 kg/(m^2^ h) and a separation factor of 239,760–39,996) in the pervaporation dehydration of ethanol (4–20 wt.% water) compared to other types of supported membranes without surface modification described in the literature.

The comparison of the transport properties of the SA-5HF/PAN^CaCl2^-5Lbl^PSS/SA^ membrane to the other types of membranes described in the literature for the pervaporation dehydration of tetrahydrofuran under close experimental conditions is presented in Table 9.

It was found that the developed membrane is slightly inferior to the commercial membrane CMC-VP-31 (CM Celfa) and some thin-film composite membranes (BAE-TMC/PAN and DAPE-TMC/PAN) in the pervaporation dehydration of THF (5.7 and 12 wt.% water, respectively). However, it should be noted that there is a limited number of works in which membranes are studied in the pervaporation dehydration of THF with a high water content in the feed. It should be stressed that the developed SA-5HF/PAN^CaCl2^-5Lbl^PSS/SA^ membrane was highly selective, permeable, and also stable in the case of the separation of THF/water mixtures up to 70 wt.% water in the feed (Figure 9b). 

Thus, in this work, a green and effective supported membrane based on SA with Lbl modification has been developed for efficient pervaporation dehydration, having promising potential for application in industrial dehydration processes.

## 4. Conclusions

In the present work various strategies were applied to improve the transport properties of membranes based on biopolymer sodium alginate modified by water-soluble fullerene derivatives (fullerenol and fullerene derivative with L-arginine) to improve the performance of the membrane in pervaporation dehydration, through: (I) the selection of a porous substrate; (II) the deposition of PEL layers by the Lbl method.

It was shown that the nature and structure of the porous substrate essentially influenced the pervaporation characteristics of the supported membranes. It was also demonstrated that the deposition of a thin, selective SA layer on the commercial porous substrates UPM, RC, and PAN significantly increased permeation flux by 15, 32, and 76%, respectively, compared to the dense SA membrane (0.151 kg/(m^2^ h)). Additionally, a not significant decrease of selective properties for the supported SA/UPM and SA/RC membranes (98.7 and 99.4 wt.% water in the permeate, respectively) compared to the dense SA and SA/PAN membranes (over 99.9 wt.% water in the permeate) was observed. The highest value of the PSI parameter of 19,504 kg/(m^2^ h) for the developed supported SA/PAN membrane compared to the dense SA membrane (11,072 kg/(m^2^ h)) and supported SA/UPM, SA/RC membranes (93 and 243 kg/(m^2^ h)), respectively, indicated its efficiency and high-performance in the pervaporation separation of an azeotropic water/isopropanol mixture. This was attributed to the increased porosity, the largest pore size of the skin top layer and the macrovoids in the bulk, the highest surface roughness, and the hydrophilicity of the PAN material.

The Lbl technique for the PEL deposition was successfully applied to enhance the performance of the developed PAN-supported SA membranes. To ensure the membrane’s stability during PEL coating, the developed PAN-supported membranes were subjected to cross-linking with calcium chloride before surface Lbl modification. In order to select the optimal conditions of the Lbl modification, the cross-linked supported membrane based on a pristine SA (SA/PAN^CaCl2^) was coated with three, five, and finally ten bilayers of various PEL pairs such as PSS/CS, PSS/PAH, PSS/SA, and PAA/SA. Based on the pervaporation data (the separation of an azeotropic water/isopropanol 12/88 wt.% mixture), the optimal condition of the surface modification of the SA/PAN^CaCl2^ membrane by Lbl was the deposition of five PSS/SA bilayers, which resulted in improved membrane performance compared to the SA/PAN^CaCl2^ membrane without Lbl modification: 14% increased permeation flux (0.571 kg/(m^2^ h)) and the maintenance of over 99.9 wt.% water in the permeate. The effect of the increased membrane permeability of the SA/PAN^CaCl2^-5Lbl^PSS/SA^ membrane could be related to the formation of small hydrophilic mashes in the PEL layers induced by the PEL charge density and the increased surface hydrophilicity of the modified membrane, which resulted in the improved penetration of water through the membrane in comparison with isopropanol. The casting of five PSS/SA bilayers on the SA membranes modified by fullerene derivatives further improved the properties of the membrane. It was demonstrated that the modification of the SA/PAN^CaCl2^ membrane with 5 wt.% AF and HF with the deposition of five PSS/SA bilayers increased the selectivity (over 96.3 and 99.7 wt.% water in the permeate, respectively) in the pervaporation dehydration of isopropanol (12–70 wt.% water) compared to the SA/PAN^CaCl2^-5Lbl^PSS/SA^ membrane (over 95 wt.% water in the permeate). The SA-5HF/PAN^CaCl2^-5Lbl^PSS/SA^ membrane had the highest permeation flux (0.68–1.38 kg/(m^2^ h)) compared to the SA/PAN^CaCl2^-5Lbl^PSS/SA^ (0.48–1.33 kg/(m^2^ h)) and SA-5AF/PAN^CaCl2^-5Lbl^PSS/SA^ (0.51–1.30 kg/(m^2^ h)) membranes. Thus, the most significant improvement of the membrane transport characteristics was achieved under a combination of bulk and surface modifications.

To confirm the effectiveness of the industrial application of the developed SA-5HF/PAN^CaCl2^-5Lbl^PSS/SA^ membrane, this membrane was evaluated in the pervaporation dehydration of two different alcohols (iPrOH and EtOH) and an industrially important solvent (THF) over a wide range of concentrations. It was demonstrated that the permeability of the SA-5HF/PAN^CaCl2^-5Lbl^PSS/SA^ membrane increased by 13–36% (0.68–1.38 kg/(m^2^ h)), 10–111% (0.18–1.55 kg/(m^2^ h)), and 25–58% (0.50–1.15 kg/(m^2^ h)) during the pervaporation dehydration of iPrOH (12–70 wt.% water), EtOH (4–70 wt.% water), and THF (5.7–70 wt.% water), respectively, compared to the SA/PAN^CaCl2^ membrane without Lbl modification. Additionally, the modified SA-5HF/PAN^CaCl2^-5Lbl^PSS/SA^ membrane was characterized by its high selectivity level—over 99.7, 99.0, and 89.0 wt.% water in the permeate in the pervaporation dehydration of iPrOH, EtOH, and THF, respectively. Thus, the developed SA-5HF/PAN^CaCl2^-5Lbl^PSS/SA^ membrane demonstrated its promise for industrial use in dehydration processes in the future.

## Figures and Tables

**Table 1:** Developed supported sodium alginate (SA)-based membranes.

Membrane	Content of Carbon Nanoparticles, wt.%	Cross-Linking Method	Type and Number of PEL Bilayers
SA/UPM	-	-	-
SA/RC	-	-	-
SA/PAN	-	-	-
SA-5HF/PAN	5% C_60_(OH)_22–24_	-	-
SA-5AF/PAN	5% C_60_-Arg	-	-
SA/PAN^CaCl2^	-	1.25 wt.% CaCl_2_	-
SA-5HF/PAN^CaCl2^	5% C_60_(OH)_22–24_	1.25 wt.% CaCl_2_	-
SA-5AF/PAN^CaCl2^	5% C_60_-Arg	1.25 wt.% CaCl_2_	-
SA/PAN^CaCl2^–5Lbl^PSS/CS^	-	1.25 wt.% CaCl_2_	Five bilayers of PSS/CS
SA/PAN^CaCl2^–5Lbl^PSS/PAH^	-	1.25 wt.% CaCl_2_	Five bilayers of PSS/PAH
SA/PAN^CaCl2^–5Lbl^PSS/SA^	-	1.25 wt.% CaCl_2_	Five bilayers of PSS/SA
SA/PAN^CaCl2^–5Lbl^PAA/SA^	-	1.25 wt.% CaCl_2_	Five bilayers of PAA/SA
SA-5HF/PAN^CaCl2^–5Lbl^PSS/CS^	5% C_60_(OH)_22–24_	1.25 wt.% CaCl_2_	Five bilayers of PSS/CS
SA-5AF/PAN^CaCl2^–5Lbl^PSS/CS^	5% C_60_-Arg	1.25 wt.% CaCl_2_	Five bilayers of PSS/CS

SA: sodium alginate; UPM: aromatic polysulfone amide; RC: regenerated cellulose; PAN: polyacrylonitrile; HF: polyhydroxylated fullerene (fullerenol); AF: fullerene derivative with L-arginine; PSS: poly(sodium 4-styrene sulfonate); CS: chitosan; PAH: poly(allylamine hydrochloride); PAA: polyacrylic acid; PEL: polyelectrolyte.

**Table 2:** Transport properties of the dense SA membrane and supported membranes with a thin layer based on SA, deposited on various porous substrates (UPM, RC, and PAN), in the pervaporation separation of an azeotropic water/isopropanol (12/88 wt.%) mixture at 22 °C.

Membrane	Permeation Flux, kg/(m^2^ h)	Water Content in the Permeate, wt.%	Separation Factor (*β*)	PSI, kg/(m^2^ h)
SA	0.151	>99.9	73,326	11,072
SA/UPM	0.173	98.7	536	93
SA/RC	0.200	99.4	1215	243
SA/PAN	0.266	>99.9	73,326	19,504

PSI: pervaporation separation index.

**Table 3:** The surface roughness parameters of the PAN-supported membranes based on SA and its composites with HF and AF.

Membrane	Ra, nm	Rq, nm
SA/PAN	0.7	0.9
SA-5HF/PAN	3.5	4.9
SA-5AF/PAN	10.5	14.9

**Table 4:** The surface roughness parameters, total porosity, and water permeability of the porous substrates.

Substrate	Ra, nm	Rq, nm	Total Porosity, %	Water Flux at 1 bar, L/(m^2^ h)
UPM	22.4	28.7	95.0	60
RC	21.3	25.4	87.4	200
PAN	26.5	38.8	96.5	450

**Table 5:** The surface roughness parameters for the surface-modified membranes.

Membrane	Ra, nm	Rq, nm
SA/PAN^CaCl2^-5Lbl^PSS/SA^	1.4	1.8
SA-5HF/PAN^CaCl2^-5Lbl^PSS/SA^	2.4	3.2
SA-5AF/PAN^CaCl2^-5Lbl^PSS/SA^	5.9	9.8

**Table 6:** Comparison of the transport properties of the membranes with surface modification (the deposition of PEL layers) in the pervaporation dehydration of isopropanol.

**Membranes**	**Water Content in Feed, wt.%**	**Temperature,** **°C**	**Permeation Flux,** **kg/(m^2^ h)**	**Water Content in Permeate, wt.%**	**Separation Factor,** (***β*)**	**Reference**
SA-5HF/PAN^CaCl2^–5Lbl^PSS/SA^	20	22	0.765	>99.9	39,996	This study
PVA–PAH (4.7%)/PAN–Lbl^PSS,PAH^ (ten bilayers)	20	20	0.061	99.9	3996	[27]
PVA–PAH (4.7%)/UPM–Lbl^PSS,PAH^ (ten bilayers)	20	20	0.261	68.4	9	
PVA-fullerenol (5%)-CS (20%)/UPM–Lbl^PSS,CS^ (five bilayers)	20	22	0.340	95.6	87	[40]
PVA-fullerenol (5%)-CS (20%)/UPM–Lbl^PSS,PAH^ (five bilayers)	20	22	0.282	95.5	85	
PVA-fullerenol(5%)-PAH (4.7%)/UPM–Lbl^PSS,PAH^ (ten bilayers)	20	22	0.286	98.4	246	[41]
HEC */SA-fullerenol (5%)/PAN^CaCl2^–Lbl^PSS,PAH^ (five bilayers)	20	22	0.976	92.8	52	[70]
HEC */SA-fullerenol (5%)/PAN^CaCl2^–Lbl^PSS,SA^ (five bilayers)	20	22	0.867	97.5	156	

* HEC: hydroxyethyl cellulose.

**Table 7:** Comparison of the transport properties of the SA-based and commercial PERVAP^TM^ 1201 membranes in the pervaporation dehydration of isopropanol.

Membranes	Membrane Type	Water Content in Feed, wt.%	Temperature, °C	Permeation Flux, kg/(m^2^ h)	Separation Factor, (*β*)	Reference
**SA-5HF/PAN^CaCl2^-5Lbl^PSS/SA^**	**supported**	**12**	**22**	**0.681**	**73,326**	**This study**
SA-chitosan wrapped MWCNT (2%)	dense	10	30	0.218	6419	[71]
SA-phosphomolybdic acid (10%)	dense	10	30	0.282	9028	[72]
SA-phosphotungstic acid modified by ammonium carbonate (10%)	dense	10	30	0.316	8991	[73]
SA-gelatin (10%)	dense	10	30	0.085	4277	[74]
SA-fullerenol (5%) ^CaCl2^	dense	12	22	0.240	73,326	[47]
SA-fullerenol (5%)/PAN^CaCl2^	supported	12	22	0.641	73,326	
PERVAP^TM^ 1201	supported	12	22	0.028	73,326	
HEC */SA-fullerenol (5%)/PAN^CaCl2^	supported	12	22	0.420	73,326	[70]
**SA-5HF/PAN^CaCl2^-5Lbl^PSS/SA^**	**supported**	**30**	**22**	**1.090**	**23,331**	**This study**
SA-poly(acrylamide) grafted guar gum (75/25)	dense	30	30	0.164	153	[75]
SA-polystyrene sulfonic acid-co-maleic acid	dense	30	30	~0.223	~1800	[76]
SA-aluminum with mesoporous silica (20%)	dense	30	30	0.256	∞	[77]
SA-heteropolyacids (10%)	dense	30	30	~0.263	~1260	[78]
SA-karayagum (15%)	dense	30	30	0.486	1613	[79]
SA-fullerenol (5%) ^CaCl2^	dense	30	22	0.504	11,763	[47]
SA-fullerenol (5%)/PAN^CaCl2^	supported	30	22	1.202	2331	
HEC */SA-fullerenol (5%)/PAN^CaCl2^	supported	30	22	1.212	50	[70]
SA-NGQD * (100 ppm)/PES * ^CaCl2^	supported	30	25	1.822	788	[80]
SA-OGQD * (100 ppm)/PES * ^CaCl2^	supported	30	25	1.663	2331	
SA-reduced graphene oxide (3%)/PES * ^CaCl2^	supported	30	25	~1.750	~23,000	[81]
SA-graphene quantum dots+reduced graphene oxide (3%)/PES * ^CaCl2^	supported	30	25	~1.400	~23,000	

* HEC: hydroxyethyl cellulose; PES: polyethersulfone substrate; NGQD: nitrogen-doped graphene quantum dots; OGQD: oxygen-passivated graphene quantum dots.

**Table 8:** Comparison of the transport properties of the membranes in the pervaporation dehydration of ethanol.

Membranes	Membrane Type	Water Content in Feed, wt.%	Temperature, °C	Permeation Flux, kg/(m^2^ h)	Separation Factor, (*β*)	Reference
**SA-5HF/PAN^CaCl2^-5Lbl^PSS/SA^**	**supported**	**4**	**22**	**0.185**	**239,760**	**This study**
PVA/PS * hollow fiber membrane	supported	5	50	0.06	53	[82]
**SA-5HF/PAN^CaCl2^-5Lbl^PSS/SA^**	**supported**	**12**	**22**	**0.568**	**73,326**	**This study**
Polyacrylic acid sodium-NaA zeolite/PAN	supported	10	30	0.533	436	[83]
DETA-TMC */CA *	supported	10	25	0.860	1116	[84]
DAPL-SCC */mPAN	supported	10	25	0.600	264	[85]
PAA-PA/PAN	supported	10	25	0.830	1791	[86]
PA-nanoNaX zeolite/mPAN	supported	10	25	4.500	30	[87]
30 bilayers of CS/graphene oxide on mPAN	supported	10	70	2.350	3390	[88]
SA/PFSA */ceramic	supported	15	75	0.821	5661	[89]
**SA-5HF/PAN^CaCl2^-5Lbl^PSS/SA^**	**supported**	**20**	**22**	**0.785**	**39,996**	**This study**
PVA-maleic acid/PES	supported	20	60	0.444	13	[90]
CS-PVA/PAN	supported	20	60	1.500	40	[91]
PVA-zeolite 4A (20%)/PAN	supported	23.57	60	0.936	710	[92]

* PS: polysulfone; CA: cellulose acetate; DETA: diethylenetriamine; TMC: trimesoyl chloride; DAPL: 1,3-diamino-2-propanol; SCC: succinyl chloride; PFSA: perfluorinated sulfonic acid.

**Table 9:** Comparison of the transport properties of the membranes in the pervaporation dehydration of THF.

Membranes	Membrane Type	Water Content in Feed, wt.%	Temperature, °C	Permeation Flux, kg/(m^2^ h)	Separation Factor (*β*)	Reference
**SA-5HF/PAN^CaCl2^-5Lbl^PSS/SA^**	**supported**	**5.7**	**22**	**0.510**	**1086**	**This study**
PVA-HEC	dense	5.5	30	0.082	160	[93]
PVA-HEC *-clay microfiller	dense	5.5	30	0.090	185	
PVA-HEC *-clay nanofiller	dense	5.5	30	0.112	195	
CS-NaY zeolite	dense	5	30	0.170	2092	[94]
Polyaniline	dense	4	55	0.622	36	[95]
PVA-fullerenol (5%)/UPM	supported	5.7	30	0.250	2347	[96]
CS-polyacrylonitrile/UPM	supported	5.7	35	0.202	1487	[97]
CS-polystyrene/UPM	supported	5.7	35	0.226	101	
CMC-VP-31 (CM Celfa)	supported	4	25	3.500	1976	[98]
**SA-5HF/PAN^CaCl2^-5Lbl^PSS/SA^**	**supported**	**12**	**22**	**0.807**	**342**	**This study**
BAE *-TMC/PAN	supported	10	30	1.399	2036	[99]
DAPE *-TMC/PAN	supported	10	25	1.070	8991	[100]
**SA-5HF/PAN^CaCl2^-5Lbl^PSS/SA^**	**supported**	**20**	**22**	**0.873**	**96**	**This study**
crosslinked polybenzoxazine (CRPBz)-lignin	dense	20	25	0.425	3996	[101]
PVA-fullerenol (5%)/UPM	supported	20	30	~1.000	~9	[96]
**SA-5HF/PAN^CaCl2^-5Lbl^PSS/SA^**	**supported**	**30**	**22**	**0.902**	**29**	**This study**
crosslinked polybenzoxazine (CRPBz)-lignin	dense	30	25	0.490	19,440	[101]
PVA-fullerenol (5%)/UPM	supported	30	30	~1.200	~3.5	[96]

* TMC: trimesoyl chloride; BAE: 2-bis(2-aminoethoxy)ethane; DAPE: 1,3-diaminopropane; HEC: hydroxyethyl cellulose.

## Data Availability

Data is contained within this article.
